# Brain pericytes acquire a microglial phenotype after stroke

**DOI:** 10.1007/s00401-014-1295-x

**Published:** 2014-05-22

**Authors:** Ilknur Özen, Tomas Deierborg, Kenichi Miharada, Thomas Padel, Elisabet Englund, Guillem Genové, Gesine Paul

**Affiliations:** 1 Translational Neurology Group, Department of Clinical Science, Wallenberg Neuroscience Center, Lund University, 22184 Lund, Sweden; 2 Experimental Neuroinflammation Laboratory, Department of Experimental Medical Science, BMC, Lund University, 22184 Lund, Sweden; 3 Department of Molecular Medicine and Gene Therapy, Lund Strategic Center for Stem Cell Biology and Cell Therapy, BMC, Lund University, 22184 Lund, Sweden; 4 Department of Neuropathology, Scania University Hospital, 22185 Lund, Sweden; 5 Division of Vascular Biology, Department of Medical Biochemistry and Biophysics, Karolinska Institute, 17177 Stockholm, Sweden; 6 Department of Neurology, Scania University Hospital, 22185 Lund, Sweden

**Keywords:** Pericytes, Stroke, Microglia, Human brain, Regulator of G-protein signaling 5

## Abstract

Pericytes are located on the abluminal side of endothelial cells lining the microvasculature in all organs. They have been identified as multipotent progenitor cells in several tissues of the body including the human brain. New evidence suggests that pericytes contribute to tissue repair, but their role in the injured brain is largely unknown. Here, we investigate the role of pericytes in ischemic stroke. Using a pericyte-reporter mouse model, we provide unique evidence that regulator of G-protein signaling 5 expressing cells are activated pericytes that leave the blood vessel wall, proliferate and give rise to microglial cells after ischemic brain injury. Consistently, we show that activated pericytes express microglial markers in human stroke brain tissue. We demonstrate that human brain-derived pericytes adopt a microglial phenotype and upregulate mRNA specific for activated microglial cells under hypoxic conditions in vitro. Our study indicates that the vasculature is a novel source of inflammatory cells with a microglial phenotype in brain ischemia and hence identifies pericytes as an important new target for the development of future stroke therapies.

## Introduction

The central nervous system (CNS) has, apart from the retina, the highest density of pericytes [49]. Besides playing an important role in angiogenesis and in the control of the blood brain barrier [3, 6], pericytes have been shown to react to tissue injury. Pericytes are known to replace tissue-specific cells such as odontoblasts [19], myocytes [13], myofibroblasts [29] and adipocytes [50] or indirectly mediate repair processes [9, 17, 40].

The response of pericytes to CNS injury in vivo is largely unknown. In the spinal cord, a subtype of pericytes contributes to scar formation [23]. However, pericytes may respond differently to specific injuries of certain tissues since they constitute a heterogeneous cell population.

In ischemic stroke, a prolonged reduction in blood flow can lead to blood–brain barrier dysfunction, edema, inflammation, and neuronal as well as glial cell death [14]. Endogenous repair mechanisms are important to understand to develop therapies for neuroprotection and brain repair to improve neurological function in stroke patients. Since brain pericytes actively control a variety of functions such as microcirculation, angiogenesis, and a functional blood brain barrier [3, 6] these cells are likely to play an important role during brain ischemia and in the post-ischemic period.

We therefore sought to investigate the response of brain pericytes to ischemic stroke. We identify pericytes using a knock-out/knock-in reporter-labeling approach by utilizing mice where green fluorescent protein (GFP) is expressed under the regulator of G-protein signaling 5 (*Rgs5*) [39]. Regulator of G-protein signaling 5 is a gene solely expressed in pericytes [8, 10], particularly in immature pericyte progenitors and activated pericytes [7].

We show for the first time that in response to focal ischemic brain injury, pericytes are activated, proliferate, leave the blood vessel wall and migrate into the adjacent ischemic brain parenchyma where they adopt an ameboid morphology and express microglial markers. In addition, we show in human stroke that pericytes express RGS5 and co-express the macrophage/microglial marker galectin-3 (GAL-3). Using immunocytochemistry and qPCR, we demonstrate that human pericytes acquire a microglial phenotype upon exposure to hypoxic conditions in vitro. Our data suggest that pericytes may constitute a previously unknown vascular source of microglial cells in stroke and might therefore play a role in the pathogenesis of tissue damage after brain ischemia.

## Materials and methods

### Animals

The in vivo study was conducted using reporter *Rgs5* ^*gfp/*+^ mice [39]. In this mouse, GFP is expressed in the cytoplasm under the pericyte-specific RGS5 promoter which makes it possible to track pericytes. The *Rgs5* ^*gfp/*+^ mouse we use is a knock-out/knock-in mouse and is retaining the full promoter region [39]. Mice were housed under standard conditions, with free access to water and food. All animal experiments followed “Principles of laboratory animal care” (NIH publication No. 86-23, revised 1985) and experiments were approved by the Ethical Committee at Lund University (M90-12 and M479-12).

### Permanent middle cerebral artery occlusion (pMCAO)

Focal cerebral ischemia was induced by permanently occluding the distal part of the left middle cerebral artery (MCA) as previously described [26]. In brief, the mice were placed on an operating table at 37.5 °C under a dissecting microscope. Under isoflurane anesthesia, an incision was made between the left lateral part of the orbit and the left ear. The parotid gland and the temporal muscle were displaced in the distal direction with a pair of anatomical forceps. A small craniotomy was made with a 0.8-mm burr above the anterior distal branch of the MCA using a high-speed microdrill. The MCA was exposed and occluded permanently by electrocoagulation using an electrosurgical unit (ICC50; Erbe, Germany). Marcain was used as local analgesia before suturing the wound.

### BrdU administration

Mice were injected intraperitoneally for 2 weeks with bromodeoxyuridine (BrdU, 50 mg/kg, Sigma-Aldrich) twice daily, starting 2 h after injury.

### Human tissue

Human brain tissues were obtained from patients who suffered from stroke and age-matched controls for this study. Before the investigation, the entire collection of brain sections, was subjected to a neuropathological whole-brain analysis for clinical diagnostic purposes, according to routine procedures at the Department of Pathology, Division of Neuropathology, Lund University Hospital. Human brain pericyte lines were previously derived from brain biopsies as described [43].

All procedures involving human tissue were performed with informed written consent by the patient for the donation of brain tissue. The use of human brain tissue was approved by the Regional Ethical Review Board in Lund, Sweden (Dnr 196/2010) and according to the declaration of Helsinki.

### Immunohistochemistry

Mice were killed at 1, 7 and 21 days after pMCAO and transcardially perfused with phosphate buffered saline (PBS) followed by 4 % paraformaldehyde (PFA), placed in 30 % sucrose in PBS and sectioned in the coronal plane at 40 μm. Sections were incubated with blocking solution (5 % normal donkey serum in PBS, with 0.25–2.5 % Triton-X100) for 1 h at room temperature (RT), then incubated at 4 °C or RT overnight with primary antibodies diluted in 3 % normal donkey serum. For BrdU detection, sections were pre-treated with 2 N HCl at room temperature for 30 min before staining. The following primary antibodies were used: alpha smooth muscle actin (α-SMA) (1:100, rabbit, Abcam), GFP (1:1,000, chicken, Abcam), PECAM-1 (CD31) (1:400, rat, BD), BrdU, (1:200, rat, Accurate), CD11b (1:1,000, rat, Sigma), CD13 (1:200, rat, Serotec), CD45 (1:100, rat, BD), CD45 (1:100, mouse, BD), CD68 (1:100, mouse, DAKO), collagen-IV (1:200, rabbit Serotec), fibronectin (1:300, rabbit, Abcam), fibroblast surface protein (FSP) (1:100, mouse, Sigma), galectin-3 (1:500, rat, kindly provided by Hakon Leffler), laminin (1:200, rabbit, Abcam), NG2 (1:500, rabbit, Chemicon), IBA1 (1:2,000, rabbit, Wako), anti-Ki67 (1:100, rabbit, Novacostra), anti-PDGFRβ (1:200, rabbit, cell signaling), RGS5 (1:200, mouse, ORIGENE). After washing, antibody staining was revealed using species-specific fluorophore-conjugated (Daylight 488, Cy3, Cy5 from Jackson, Alexa 488 from molecular probes) or biotin-conjugated secondary antibodies (Invitrogen). Biotinylated secondary antibodies were revealed using the ABC kit (Vector labs). DAPI (1 μg/mL, Sigma) and TOTO-3 iodide 642/660 (1:500, Invitrogen) were used for counterstaining. Control sections were stained with secondary antibody alone.

For immunocytochemistry, cells were plated on glass coverslips that were coated with vitronectin 0.50 μg/mL. Cultured cells were fixed with 2 % PFA at RT, washed in PBS and incubated for 1 h in PBS, 5 % donkey serum. Cultured cells were then stained for both primary and secondary antibodies in the presence of 0.1 % Triton X-100 and 2 % donkey serum.

### Image processing and cell counting

Immunofluorescent stainings were visualized using an epifluorescence microscopy system (Olympus BX53) and processed using cellSens digital imaging software. Confocal microscopy was performed using a Zeiss LSM510 confocal microscope (Carl Zeiss) equipped with a GreNe and a HeNe laser, using the following lines of excitation: 488, 594, and 647 nm. Figures were composed using Photoshop CS5 software. To quantify the number of GFP^+^ pericytes over time, GFP^+^ cells were counted in both, the infarct area and the corresponding contralateral area using cellSens digital imaging software (3–4 optical fields of 0.18 mm^2^ for each mice, bregma −1.2 to 1.5 mm, *n* = 5, per group). To determine proliferation in RGS5-expressing pericytes, the number of GFP^+^ cells and GFP^+^/BrdU^+^ cells was counted on three 40× confocal pictures spanning randomly the infarct area including both infarct core and peri-infarct area 7 days after pMCAO. Approximately 200 cells were counted per field in the ipsilateral part of stroke brains. The percentage of BrdU/GFP^+^ cells was determined for each animal and the average for five animals was calculated. To quantify GFP^+^/CD11b^+^, GFP^+^/IBA1^+^, and GFP^+^/GAL-3^+^ cells at 7 day after stroke, Photoshop software was used. Double-labeled cells were counted on 40× confocal pictures spanning the peri-infarct area. Approximately 300 cells were counted per animal. The percentage of cells was determined for each animal and the average for three animals was calculated.

### Bone marrow isolation and flow cytometry

Bone marrow cells were flushed out from femur and tibia analyzed as central bone marrow. Emptied bones were minced with scissors. Then, the bone fragments were incubated with 3 mg/mL Type I Collagenase and 4 mg/mL of Dispase, for 15 min at 37 °C. Cells were collected, washed with PBS containing 2 % FBS, and analyzed as endosteal BM cells. Cell suspensions were incubated for 30 min with PE conjugated anti-CD45 (30-F11, BioLegend) and anti-CD11b APC (M1/70, BioLegend) in FACS buffer (PBS supplemented with 1 % BSA containing 0.05 % NaN3), washed, and suspended in the same buffer for analysis. 7-amino-actinomycin-d (7-ADD, Sigma) was added to the cells to exclude dead cells before analysis. Cells were analyzed for endogenous GFP, CD45 and CD11b using a FACSCalibur™ (BD) flow cytometer. Collected data were analyzed using Flow-Jo software (Tree Star).

For immunohistochemistry, the bone marrow was processed as previously described [38]. In brief, bones were post fixed for 2  h in 4 % PFA at 4  °C, then they were decalcified for 48  h in 250  mM EDTA pH  7.4 at 4  °C in a shaker. Decalcified bones were cryoprotected, sectioned in the coronal plane at 12 μm and stained for GFP, CD11b, and CD45. Stroke mouse brain sections were used as a positive control.

### Mouse microglia cells

Primary microglial cells were isolated from mixed glial cell cultures prepared from forebrain of postnatal day 1 (P1) *Rgs5* ^*gfp/*+^ pups as described previously [12]. In brief, upon reaching confluence (9–13 days after isolation), microglia adhering to the astrocytes monolayer were dislodged by shaking for 1 h and resuspended in Dulbecco’s modified Eagle Media (DMEM), supplemented with 1 % penicillin/streptomycin and 10 % fetal bovine serum (FBS), and plated on uncoated 6-well plates. For cell sorting, cells were incubated with anti-CD11b-Alexa 647 (BD) and 7-AAD sorted by FACS (FACSAria; Becton–Dickinson) or DIVA software (Becton–Dickinson) using a low stream speed to ensure a high level of cell survival. Sorted microglia cells were then exposed to oxygen–glucose deprivation (OGD) for 1 h.

### Cell culture, oxygen–glucose deprivation (OGD) and cell differentiation

Human brain pericytes were prepared according to the method as described previously [43]. In brief, tissue samples were stored in Leibowitz-15 media (Invitrogen) at 4 °C, cut and enzymatically digested in enzyme solution (Collagenase 1 mg/mL (Sigma); Dispase 1.6 mg/mL (Roche); Trypsin 0.25 mg/mL (Sigma); DNase I 80 U/mL (Sigma) in DMEM and 4.5 mg/mL glucose (Invitrogen) at 37 °C for 20 min. Cells were plated on 24-well culture dishes and incubated at 37 °C/5 % CO_2_ in DMEM/F-12/Glutamax/B27 (Invitrogen). Sorted cells were maintained in culture medium consisting of Stemline medium (Sigma-Aldrich), 2 % FBS, 1 % penicillin/streptomycin and 1 % Glutamax. For flow cytometry, cultured cells at high passages were labeled with the following commercial antibodies: anti-CD140b-PE anti-CD13-PECY7 anti-CD11b-PE, anti-CD14-FITC, and CD45-APC (all from BD). For OGD, the cells were first rinsed with PBS and OGD was induced by a deoxygenated basic salt solution without glucose, in a hypoxic chamber (oxygen below 1  mm Hg; Electrotek, Shipley, UK) at 37 °C. After 2 h, the basic salt solution was removed and replaced by differentiation medium consisting of DMEM (Gibco Laboratories, USA), 1 μg/mL fibronectin (Sigma), 5 ng/mL basic fibroblast growth factor (bFGF) (R&D), 50 nM cAMP (Sigma) and cells were placed immediately back into the incubator (5 % CO_2_) at 37 °C. The cells were collected after 4 days for qPCR analysis or immunocytochemistry.

### Quantitation of gene expression by qPCR

Cells were homogenized in RNeasy mRNA kit (Qiagen) and stored at −80 °C before total RNA extractions according to the manufacturer’s protocol. cDNA synthesis was performed using Script™cDNA Synthesis Kit (Bio-Rad). cDNA was analyzed using real-time PCR SsoAdvanced™ SYBR^®^ Green Supermix from Bio-Rad using appropriate primers and run on a Bio-Rad CFX96 real-time quantitative PCR (qPCR) system. Gene expression was normalized to the housekeeping gene GAPDH and calculated using the 2^−ΔCt^ method. Melt curve analyses were performed to ensure the specificity of qPCR product. Primer sequences can be provided on request. Values are mean ± SEM of three independent experiments, and within each experiment, triplicate samples were assessed.

### Data analysis

Data were analyzed using GraphPad Prism version 5.04 software. Results of cell counts are reported as mean ± SD and gene expression values are expressed as mean ± SEM. The significance of differences between means was assessed by student *t* test or ANOVA with *p* < 0.05 considered statistically significant.

## Results

### RGS5 expression in pericytes

**Fig. 1:**
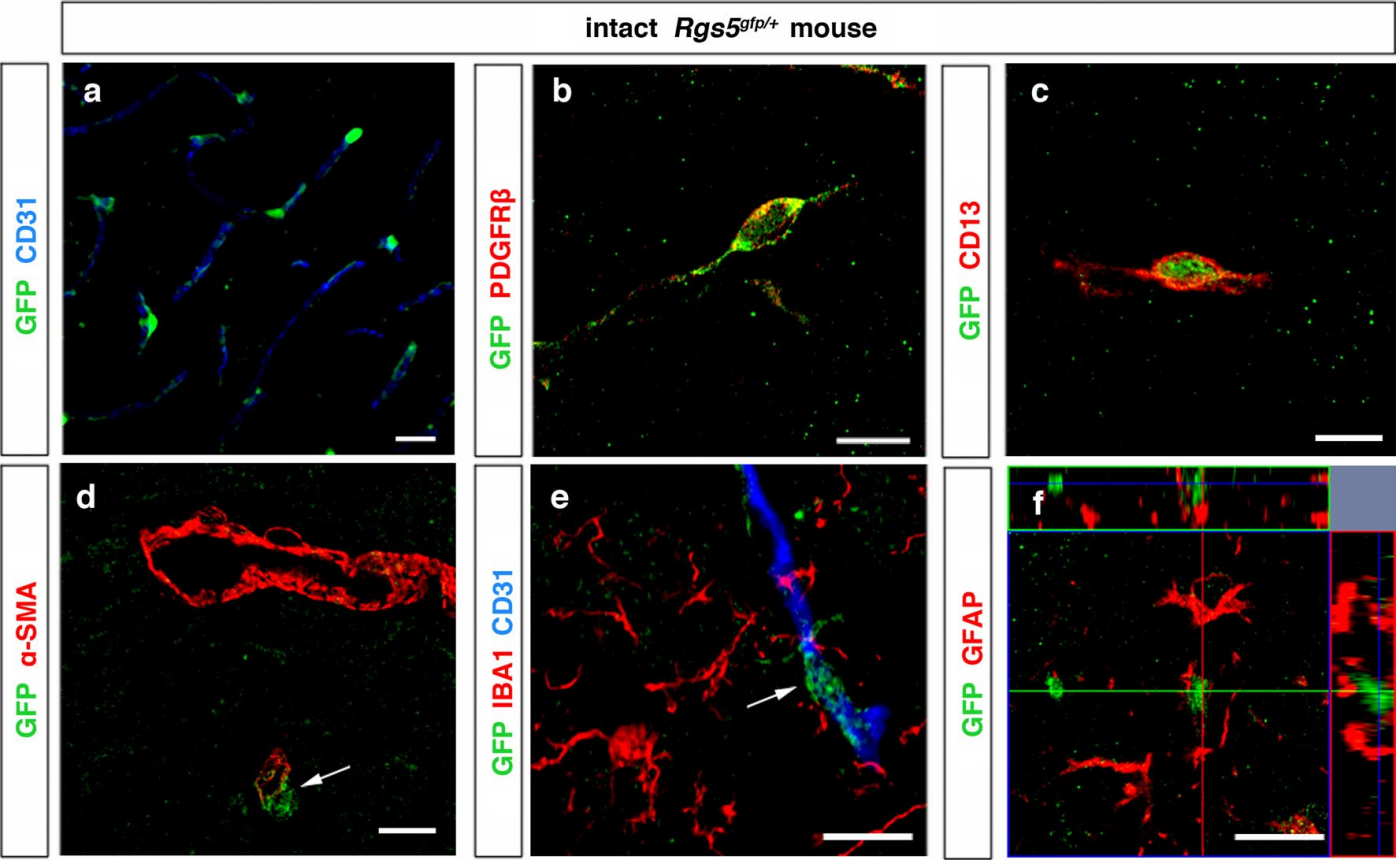
RGS5-expressing cells in the intact mouse cortex. **a** Confocal images showing cortical brain section derived from intact *Rgs5* ^*gfp/*+^ mice with blood vessel (CD31, *blue*) surrounded by GFP^+^ pericytes, *scale bar* 20 μm. GFP^+^ pericytes express PDGFRβ (*red*) (**b**), CD13 (*red*) (**c**), but not α-SMA (*red*, *arrow*) (**d**), *scale bar* 10 μm. GFP^+^ pericytes do not double-label with the microglia marker IBA1 (*red*, *arrow*) (**e**), and the astrocyte marker GFAP (*red*) (**f**), *scale bar* 10 μm

We analyzed brain section of control animals from *Rgs5* ^*gfp/*+^ mice. GFP^+^ pericytes were located around microcapillaries (Fig. 1a) where they co-expressed the pericyte marker platelet-derived growth factor receptor β (PDGFRβ) and CD13 (Fig. 1b, c), but not α-smooth muscle actin (α-SMA), a marker that was typically expressed by larger vessels (Fig. 1d). We did not detect GFP expression in any other cell type including microglia or astrocytes (Fig. 1e, f). Therefore, we could confirm previous reports that GFP is solely and exclusively expressed in pericytes in *Rgs5* ^*gfp/*+^ mice [39].

### Pericytes are activated and proliferate in response to experimental stroke

**Fig. 2:**
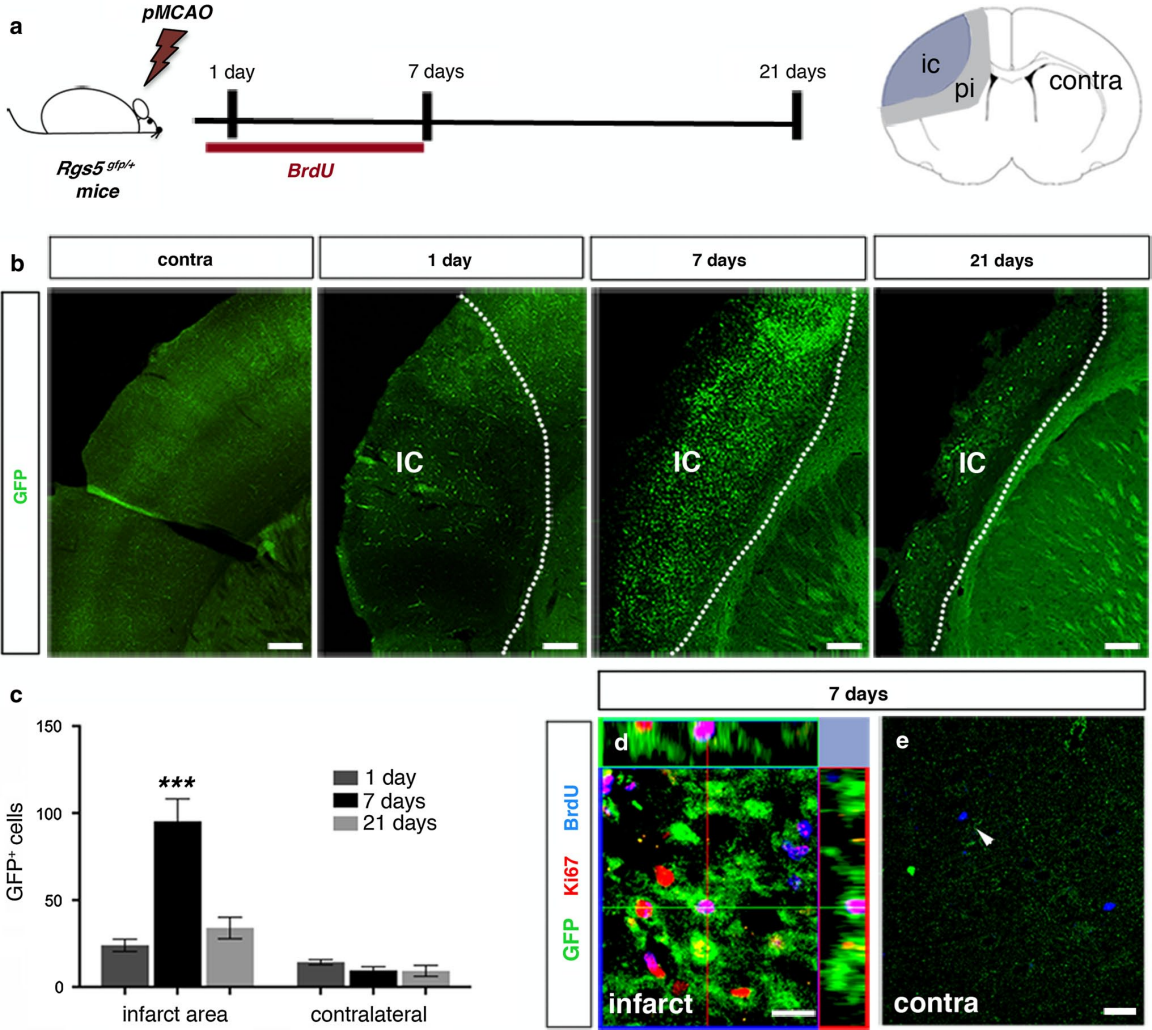
Pericytes proliferate in response to experimental stroke. **a** The illustration shows the experimental design for permanent middle cerebral artery occlusion (pMCAO) (*left*). Schematic representation of a coronal brain section showing the location of the cortical infarct core, peri-infarct area, and contralateral hemisphere (*right*). **b**, **c** GFP^+^ pericytes increase in the infarct area over time with a maximum at 7 days, *scale bar* 100 μm (*n* = 5, mean ± SD, ***p < 0.0001, ANOVA). **d** GFP^+^ pericytes express both BrdU (*blue*) and Ki67 (*red*) in the infarct area at 7 days after stroke, *scale bar* 10 μm. **e** GFP^+^ pericytes do not label with BrdU or Ki67 in the contralateral hemisphere (*arrowhead*), *scale bar* 10 μm. *pMCAO* permanent middle cerebral artery occlusion, *ic* infarct core, *pi* peri-infarct area, *contra* contralateral hemisphere

Next, we induced permanent focal brain ischemia [25] in *Rgs5* ^*gfp/*+^ mice (Fig. 2a). The ischemic injury induced an almost twofold increase in the number of GFP^+^ cells in the infarct area as compared to the non-injured contralateral side 1 day after ischemia (24.33 ± 3.6 and 14.33 ± 0.88, respectively, *n* = 5). The number of GFP^+^ pericytes peaked reaching a tenfold increase at day 7 (95.33 ± 7.42 and 9.66 ± 1.20, *n* = 5), and then gradually decreased by 21 days (34.00 ± 3.30 and 9.33 ± 1.85, *n* = 5) (Fig. 2b, c).

We then analyzed BrdU incorporation in the GFP^+^ cell population to address whether the increase in the number of pericytes is due to cell proliferation. BrdU immunolabeling revealed that proliferation of GFP^+^ pericytes started during the first days after stroke. At 7 days following the ischemic injury, 74 % ± 8.99 of the GFP^+^ pericytes had incorporated BrdU, indicating a significant increase in mitotic activity. GFP^+^ pericytes incorporating BrdU also expressed Ki67, a nuclear marker that is strictly associated with cell proliferation (Fig. 2d). None of the pericytes in the contralateral side incorporated BrdU or expressed Ki67 at any time point after the ischemia, indicating that pericytes in the adult brain remain quiescent under physiological conditions (Fig. 2e).

**Fig. 3:**
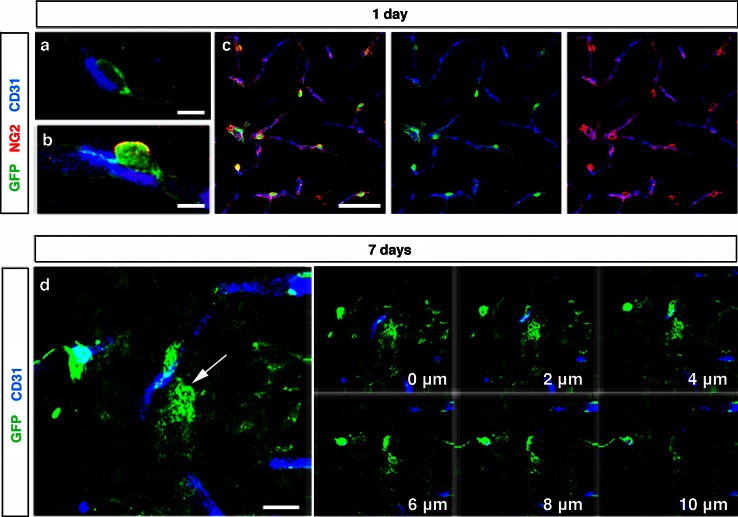
Pericytes are activated and leave the capillary wall after experimental stroke. **a** Confocal images showing GFP^+^ pericyte around blood vessel (CD31, *blue*) in the quiescent state in the contralateral hemisphere, *scale bar* 5 µm. **b** An activated pericyte (*green*) with prominent soma along the capillary (CD31, *blue*) expressing NG2 (*red*) in the infarct area, 1 day after stroke, *scale bar* 5 μm. **c** All GFP^+^ pericytes express NG2 (*red*) in the infarct area at 1 day, *scale bar* 20 μm. **d** Projection of a confocal stack showing a GFP^+^ pericyte (*arrow*) that leaves the blood vessel (CD31, *blue*) in the infarct core, 7 days after injury, *scale bar* 10 μm

Both, in the brain of intact mice and in the contralateral hemisphere of stroke mice, pericytes showed low GFP expression and a flat morphology consistent with a quiescent state [39] (Fig. 3a). One day after the injury, GFP^+^ cells were still found in close proximity to blood vessels in the infarct area. However, they displayed an activated morphology with a prominent cell body and elongated processes as previously described [15] (Fig. 3b). To further elucidate that GFP^+^ cells in the infarct area are activated pericytes, we stained sections with an antibody for Neuron-Glial 2 chondroitin sulfate proteoglycan (NG2). NG2 is expressed on the surface of activated pericytes during the development and in the adult under both physiological and pathological conditions [41, 42, 47]. All GFP^+^ pericytes associated with capillaries were positive for NG2 at 1 day after the injury (Fig. 3b, c).

Seven days post-injury, GFP^+^ cells had left the microcapillary wall and migrated into the surrounding parenchyma (Fig. 3d). GFP expression remained in cells that had migrated into the parenchyma at day 7, but was weaker compared to day 1. GFP^+^ cells that had lost contact with blood vessels displayed an ameboid morphology (Fig. 3d), similar to the morphology that has been described for pericytes leaving the blood vessels in response to traumatic brain injury [16]. We therefore decided to further investigate the phenotype of the parenchymal GFP^+^ cells to elucidate whether pericytes give rise to another cell type.

### Pericytes express microglial markers after experimental stroke

**Fig. 4:**
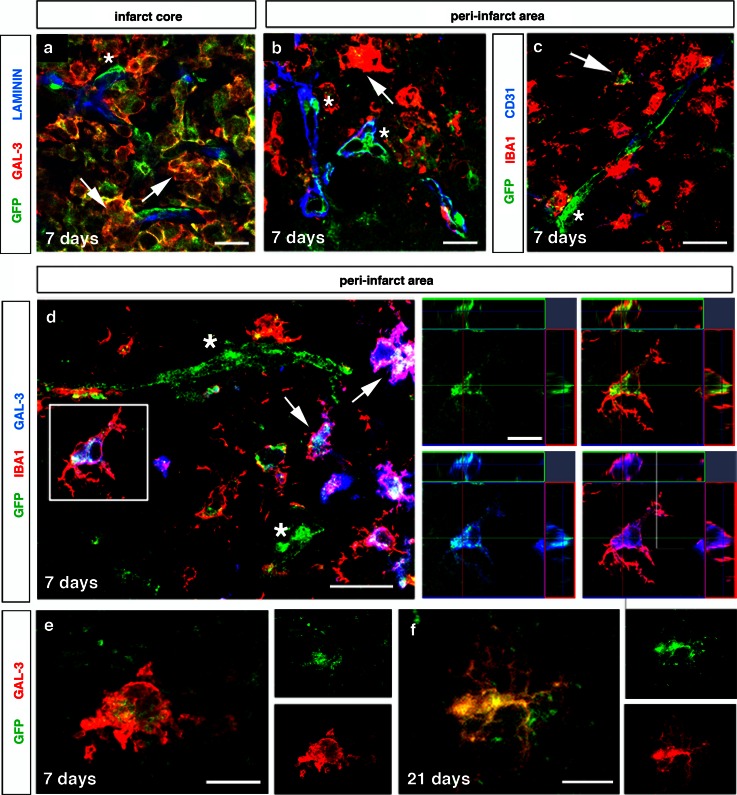
Pericytes express microglia markers GAL-3 and IBA1 after experimental stroke. Seven days after the ischemic injury, GFP^+^ cells in the parenchyma co-express GAL-3 (*red*, *arrows*), whereas GFP^+^ pericytes located around the blood vessels (laminin, *blue*) are negative for GAL-3 (*red*, *asterisk*) in **a** the infarct core and **b** the peri-infarct area, *scale bar* 20 μm. **c** GFP^+^ cells co-labeling with IBA1 (*red*, *arrow*) and GFP^+^ pericytes around a blood vessel (CD31, *blue*) negative for IBA1 (*asterisk*) in the peri-infarct area, *scale bar* 10 μm. (**d**) GFP^+^ cells, with characteristics of either ameboid (*arrows*) or ramified microglia (framed cell) in the parenchyma, co-expressing both GAL-3 (*blue*) and IBA1 (*red*), and activated GFP^+^ pericytes negative for GAL-3 and IBA1 (*asterisk*) in the peri-infarct area, 7 days after ischemic injury, *scale bar* 20 μm. *Right panels* show high-magnification confocal images of a ramified GFP^+^ cell co-expressing GAL-3 (*blue*) and IBA1 (*red*, framed cell), *scale bar* 10 μm. High magnification of GAL-3-expressing (*red*) GFP^+^ cell in the peri-infarct area 7 days (**e**) and 21 days (**f**) after the injury, *scale bar* 10 μm

Pericytes have been suggested to have phagocytic activities in vitro [5] and to give rise to follicular dendritic cells in lymphoid tissue in vivo [34]. To examine whether pericytes give rise to microglial cells in stroke, we stained brain sections for the microglial markers GAL-3, IBA1 and CD11b [30, 36]. In the infarct area, parenchymal GFP^+^ cells expressed GAL-3 and IBA1, whereas pericytes still in contact with microvessels were negative for these microglial markers at 7 days after the injury (Fig. 4a–c). GFP^+^ cells in the parenchyma had an ameboid and intermediate form as characteristic of activated microglia/macrophages or an arborized shape (Fig. 4d–f).

**Fig. 5:**
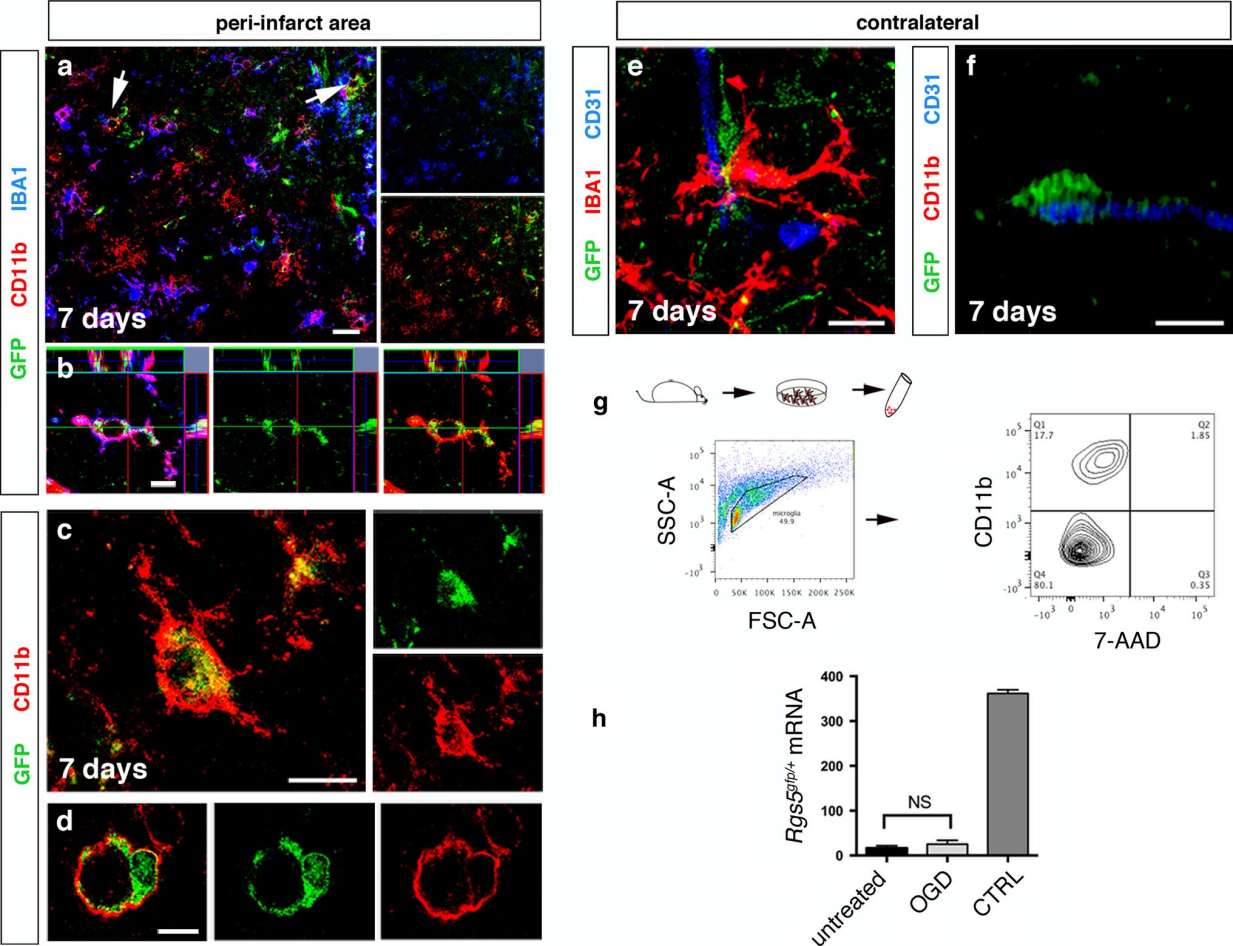
Pericytes express microglia marker CD11b after stroke. **a** GFP^+^ cells co-localized with both IBA1 (*blue*) and CD11b (*red*, *arrows*) in the peri-infarct area, 7 days after injury, *scale bar* 20 μm. High-magnification confocal image of CD11b-expressing (*red*) GFP^+^ cells in the parenchyma (**b**, **c**) and around the blood vessel (**d**) in the peri-infarct area, 7 days after injury, *scale bars* 10 μm. Quiescent GFP^+^ pericytes around the blood vessel (CD31, *blue*) do not label with microglia markers IBA1 (*red*) (**e**) and CD11b (*red*) (**f**) in the contralateral side of the ischemic brain, *scale bar* 10 μm. **g** Microglia cells derived from *Rgs5* ^*gfp/*+^mice were positively sorted for the microglia marker CD11b. **h** There was no upregulation of *Rgs5* mRNA levels in OGD-stimulated microglia derived from *Rgs5* ^*gfp/*+^mice. *Rgs5* ^*gfp/*+^mouse brain served as a positive control (*p* = 0.2292, two-tailed *t* test). *OGD* oxygen–glucose deprivation, *CTRL* control, *NS* not significant

We then quantified GFP^+^ cells in the peri-infarct area. At 7 days after stroke, 75 % ± 5.5 of GFP^+^ cells expressed GAL-3 (Fig. 4d). GFP^+^ cells also co-labeled with CD11b (Fig. 5a–d). At 7 days, 88 % ± 6.1 of GFP^+^ cells expressed CD11b. Similar to GFP^+^/GAL-3^+^ cells, GFP^+^/CD11b^+^ had the morphology of activated microglia (Fig. 5b, c) and co-localized with IBA1 (Fig. 5b). Interestingly, some activated pericytes that still localized around the capillaries were positive for CD11b (Fig. 5d). In the peri-infarct area, 44 % ± 30.08 of GFP^+^ cells expressed IBA1.

Out of the microglial population, 25.6 % ± 2.5 of the GAL-3^+^ cells, 49 % ± 4.5 of CD11b^+^ cells and only 16 % ± 11.7 of IBA1^+^ cells were positive for GFP at 7 days after stroke suggesting that only a limited number of microglia were derived from pericytes. We did not observe any expression of IBA1, CD11b (Fig. 5e, f) and GAL-3 (data not shown) in quiescent GFP^+^ pericytes in the contralateral side of the stroke brains or in intact mice.

We also tested if *Rgs5* mRNA is upregulated in stimulated microglial cells and thus may lead to unspecific GFP expression in activated microglia. We isolated microglial cells from *Rgs5* ^*gfp/*+^ mice and sorted them positively for CD11b (Fig. 5g). We then exposed these microglial cells to ischemic conditions in vitro, using OGD, and analyzed the activated microglia using qPCR. We did not detect any upregulation of *Rgs5* mRNA levels in activated microglia upon OGD exposure (Fig. 5h).

**Fig. 6:**
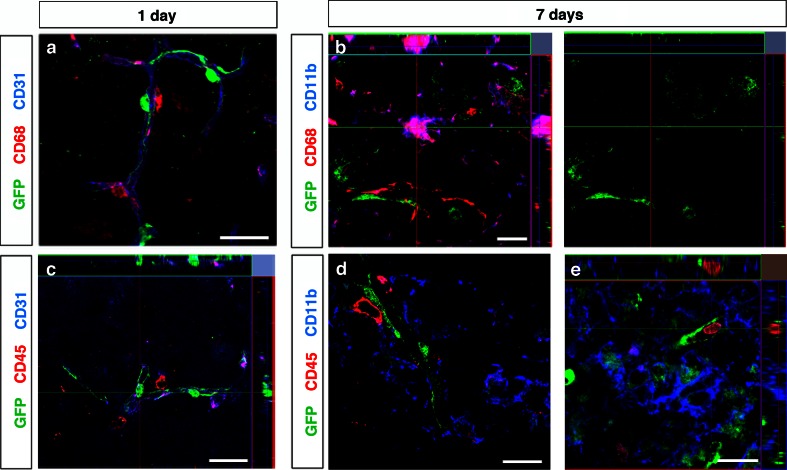
Pericytes are negative for the macrophage/microglial marker CD68 and the leukocyte marker CD45. **a** A confocal image showing that CD68^+^ cells (*red*) are closely associated with blood vessels (CD31, *blue*), but do not co-label with GFP^+^ pericytes in the infarct core 1 day after stroke, *scale bar* 20 μm. **b** Seven days after stroke, CD68^+^/CD11b^+^ cells are seen but do not co-localize with GFP^+^ cells, *scale bar* 10 μm. **c** A confocal image showing that CD45^+^ cells (*red*) are found closely associated with blood vessels (CD31, *blue*), but do not co-label with GFP^+^ pericytes in the infarct core 1 day after stroke, *scale bar* 10 μm. **d**, **e** Seven days after stroke, CD45^+^/CD11b^+^ cells do not co-localize with GFP^+^ cells, *scale bars* 20 μm

We next examined pericytes in the infarct area for co-expression of CD68, a macrophage/microglia marker. CD68^+^ cells were found closely associated to blood vessels and in the parenchyma of the infarct area. Perivascular CD68^+^ cells had a similar morphology to pericytes but were negative for GFP, suggesting that these cells may be perivascular macrophages [4, 24] (Fig. 6a). Seven days after the injury, CD68^+^ cells stained positive for CD11b, but none of the CD68^+^/CD11b^+^ cells overlapped with GFP^+^ pericytes (Fig. 6b). We also examined GFP^+^ pericytes for co-expression of CD45 in the infarct area. Cells only positive for CD45 were found close to pericytes at 1 and 7 days following injury; however, we could never detect CD45^+^ cells that co-labeled with GFP (Fig. 6c–e).

### RGS5-expressing pericytes are not activated in the bone marrow in response to experimental stroke

**Fig. 7:**
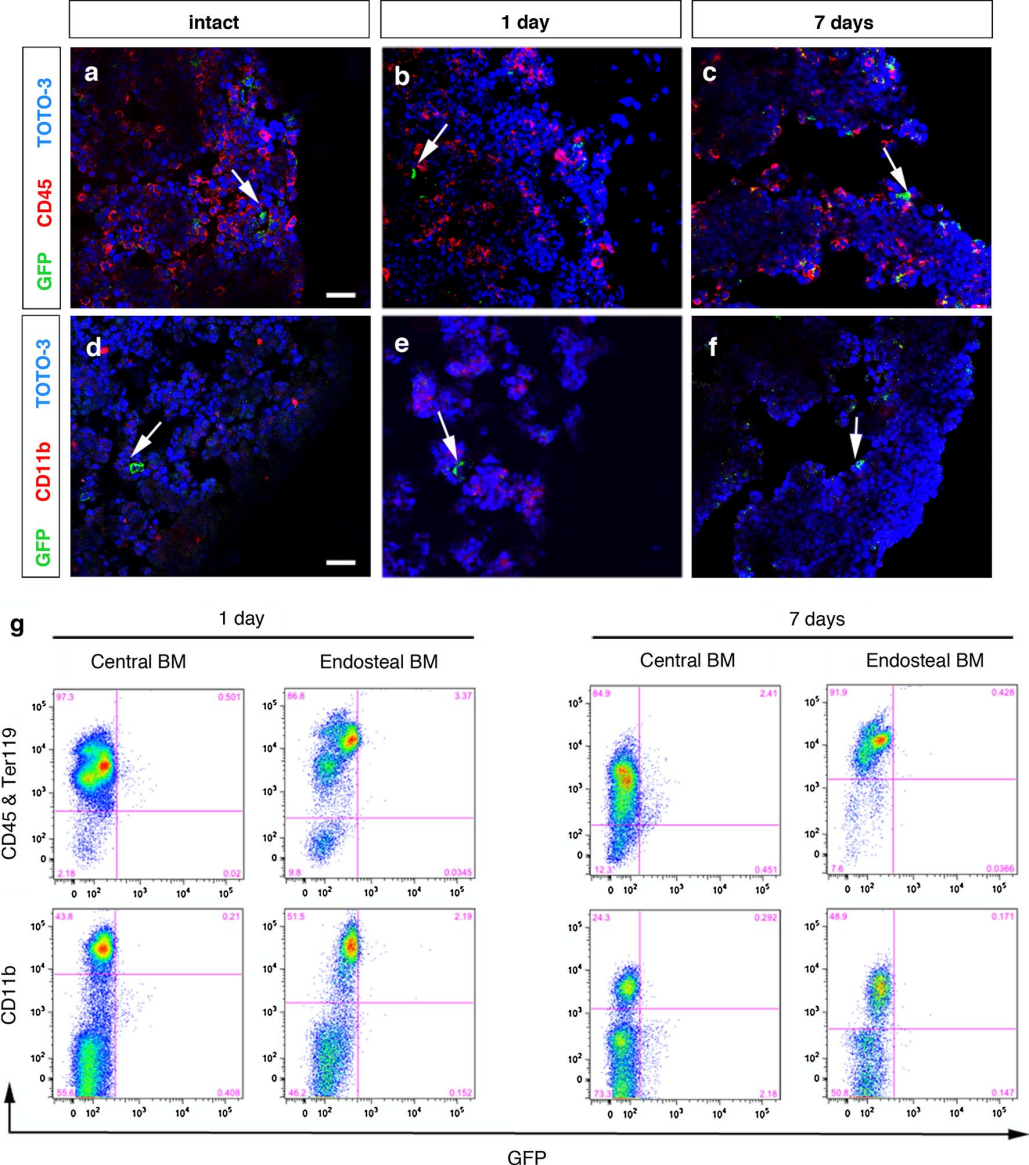
Pericytes are not activated in the bone marrow after stroke. **a**–**f** Confocal images showing low frequency of GFP^+^ cells found in the bone marrow (*arrows*). GFP^+^ cells do not co-localize with CD45 (*red*) in the bone marrow of intact *Rgs5* ^*gfp/*+^mice (**a**), at 1 day (**b**) and 7 days (**c**) after stroke, *scale bar* 20 μm. GFP^+^ cells do not co-localize with CD11b (*red*) in the bone marrow of intact *Rgs5* ^*gfp/*+^mice (**d**), at 1 day (**e**) and 7 days (**f**) after stroke, *scale bar* 20 μm. TOTO-3 Iodide (*blue*) was used as a nuclear counterstain. **g** Flow cytometry analysis shows that bone marrow cells isolated from *Rgs5* ^*gfp/*+^ mice 1 and 7 days after stroke are negative for GFP, but are positive for CD45 and Ter119 and CD11b. *BM* bone marrow

Next, we investigated whether GFP^+^ pericytes had infiltrated the stroke area from the bone marrow as it had been previously suggested in stroke [33]. First, we analyzed the bone marrow of intact *Rgs5* ^*gfp/*+^mice. GFP^+^ pericytes were found around capillaries in the bone marrow, albeit at low frequency (Fig. 7a, d). When we analyzed the bone marrow of *Rgs5* ^*gfp/*+^ mice at 1 and 7 days post-stroke, we did not detect any increase in GFP^+^ cells. GFP^+^ cells did not express CD45 or CD11b at 1 (Fig. 7b, c) and 7 days (Fig. 7e, f) after stroke. In addition, we also harvested both, central and endosteal bone marrow from stroke-injured *Rgs5* ^*gfp/*+^ mice at day 1 and day 7 and analyzed the bone marrow using flow cytometry. Consistent with our immunohistochemical results, we did not observe any GFP^+^ cells that expressed CD45 or CD11b (Fig. 7g).

### RGS5-expressing pericytes are distinct from scar-forming pericytes

**Fig. 8:**
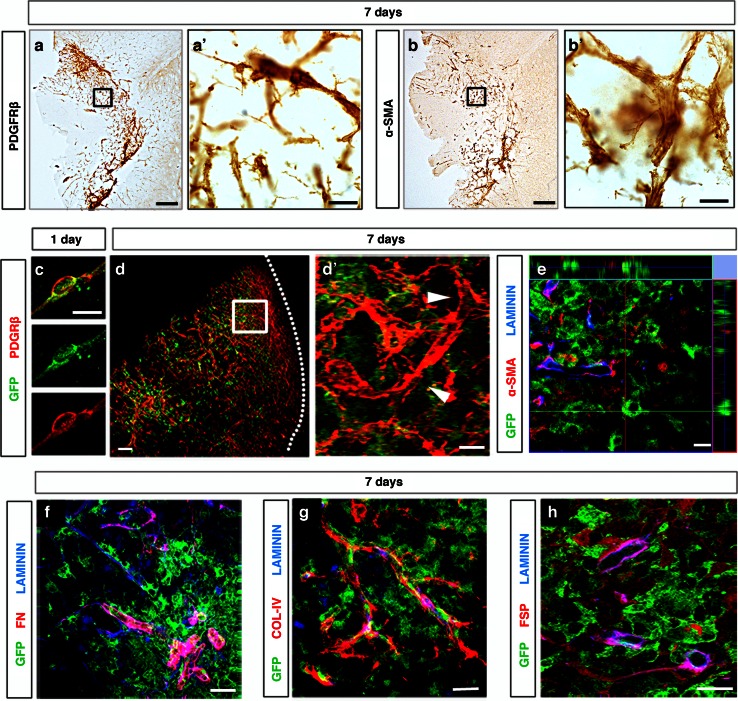
RGS5^+^ pericytes are distinct from scar-forming cells. **a** PDGFRβ expression increased around the infarct core 7 days after stroke, *scale bar* 200 μm. **a′** High magnification of PDGFRβ^+^ cells with irregular thin processes (framed area in **a**), *scale bar* 20 μm. **b** α-SMA expression around the infarct core 7 days after ischemic injury, *scale bar* 200 μm. **b′** Similar to PDGFRβ staining, α-SMA^+^ cells have irregular thin processes (framed area in **b**), *scale bar* 20 μm. **c** GFP^+^ cells express PDGFRβ (*red*) 1 day after injury, *scale bar* 10 μm. **d** GFP^+^ cells that have left the capillary wall are negative for PDGFRβ (*red*) in the infarct core (*dotted area*) 7 days after the injury, *scale bar* 10 μm. **d′** PDGFRβ-expressing cells (*red*) do not co-localize with GFP^+^ cells (*arrowheads*) (framed area in **d**), *scale bar* 10 μm. GFP^+^ cells do not express **e** α-SMA (*red*) and fibrous extracellular matrix proteins including **f** fibronectin (*red*), **g** collagen-IV (*red*) or **h** fibroblast-specific protein (*red*) in the infarct core 7 days after the injury, *scale bars* 20 μm. *FN* fibronectin, *col-IV* collagen-IV, *FSP* fibroblast-specific protein

PDGFRβ^+^ pericytes have previously been shown to contribute to scar tissue formation after spinal cord injury and these particular pericytes have been named as type A pericytes [23]. We found that both PDGFRβ^+^ and α-SMA^+^ cells were mainly located in and around the infarct core (Fig. 8a, b), but displayed differences in morphology and tissue distribution compared to GFP^+^ pericytes. Although activated GFP^+^ pericytes in the infarct core were positive for PDGFRβ 1 day after the injury (Fig. 8c), at 7 days after the injury there was no overlap between parenchymal pericyte-derived cells and PDGFRβ^+^ or α-SMA^+^ cells in the infarct core (Fig. 8d, e).

Next, we examined other markers expressed in stromal cells that are associated with scar tissue formation. Interestingly, in contrast to previously described type A pericytes [23], GFP^+^ pericyte-derived cells were negative for the fibroblast markers fibronectin, collagen-IV and the fibroblast surface protein (Fig. 8f–h), suggesting that GFP^+^ pericytes are a subpopulation of pericytes that do not give rise to scar tissue in the brain following stroke.

### Human brain pericytes acquire a microglial phenotype after stroke

**Fig. 9:**
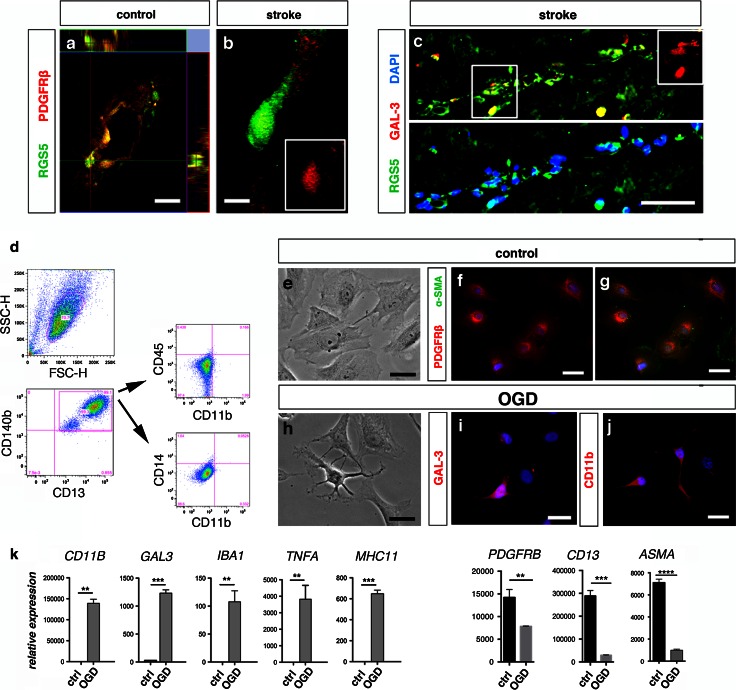
Human brain pericytes acquire a microglial phenotype. Confocal image showing, **a** neocortical brain section with a capillary lined with pericytes doubled labeled for RGS5 (*green*) and PDGFRβ (*red*), *scale bar* 10 μm, **b** RGS5^+^ activated pericyte (*green*) expressing PDGFRβ (*red*, framed) with prominent soma in the peri-infarct area of stroke human brain, *scale bar* 5 μm. **c** RGS5^+^ cells (*green*) co-express GAL-3 (*red*, framed area) around the blood vessels (DAPI, *blue*, *lower panel*) in the peri-infarct area of stroke human brain, *scale bar* 50 μm. **d** Representative histograms of flow cytometry analysis of human brain-derived pericyte line. Cells express both CD140b/PDGFRβ and CD13 (99.1 %), but no markers for microglia/macrophages (CD45 and CD11b) (0.166 %) or monocytes (CD14 and CD11b) (0.052 %). **e** Bright field image showing human brain-derived pericytes under control conditions, *scale bar* 20 μm. Human brain-derived pericytes express, **f**, **g** PDGFRβ (*red*) and α-SMA (*green*). **h** Morphological changes in the proliferating pericytes after 2 h OGD, *scale bar* 20 μm. Human brain-derived pericytes express, **i** GAL-3 and **j** CD11b after exposure to OGD, *scale bar* 20 μm. **k** *Graphs* showing upregulation of mRNA levels of microglia markers in human brain-derived pericytes stimulated with OGD compared to unstimulated controls; *CD11B* (**p < 0.004), *IBA1* (**p < 0.005), *GAL3* (***p < 0.0001), *TNFA* (**p < 0.001), and *MHC11* (***p < 0.0001). At the same time, mRNA expression for pericyte markers decreased significantly in stimulated pericytes compared to unstimulated controls; *PDGFRB* (**p < 0.0034), *CD13* (***p < 0.0001) and *ASMA* (****p < 0.0001), two-tailed *t* test. *OGD* oxygen–glucose deprivation

We next analyzed sections of human postmortem stroke brain and control samples. RGS5 staining identified pericytes that lined the microvessels and expressed PDGFRβ (Fig. 9a, b). Consistent with our results in rodents, we found that pericytes expressed RGS5 in human stroke samples and showed an activated morphology with a round cell body (Fig. 9b, c). A proportion of pericytes co-expressed RGS5 and GAL-3 along the capillaries in the peri-infarct area (Fig. 9c).

Finally, we examined whether ischemic conditions can induce pericytes to acquire a microglial phenotype in vitro. We have previously reported that human brain-derived pericytes have mesenchymal stem cell characteristics and are able to differentiate into several different cell lineages in vitro [43]. Consistent with our previous data, flow cytometry analysis showed that human-derived pericytes highly express PDGFRβ/CD140b and CD13 (99.1 %). Human-derived pericytes did not express monocyte/macrophage (CD45, CD11b; 0.166 %) or monocyte markers (CD14, CD11b; 0.0528 %) as analyzed by flow cytometry (Fig. 9d). Importantly, using qPCR, we did not detect mRNA for microglial markers such as *CD11B*, *GAL*-*3*, *IBA1*, tumor necrosis factor alpha (*TNFA*) and major histocompatibility complex class II (*MHC11*) (Fig. 9k). To mimic ischemia-like conditions in vitro, human brain-derived pericytes were exposed to OGD and analyzed using immunocytochemistry and qPCR. Pericytes had the typical flat morphology in vitro and expressed PDGFRβ and α-SMA (Fig. 9e–g). Pericytes exposed to OGD changed their morphology compared to control cultures and expressed CD11b and GAL-3 (Fig. 9h–j). mRNA levels of typical genes for microglial cells *CD11B, GAL*-*3, IBA1,* *TNFA* and *MHC11* were upregulated following OGD, but not expressed in control cells (Fig. 9k). Consistent with our findings in vivo, whilst mRNA for microglial markers was upregulated in cultures induced after OGD, the expression of the mRNA for pericyte markers *PDGFRB, CD13* and *A*-*SMA*, was significantly decreased in the differentiated cells compared to controls (Fig. 9k). These data support that ischemic conditions can induce a phenotypic switch in pericytes toward a microglial phenotype.

## Discussion

We have shown that pericytes not only strongly respond to ischemic brain injury with migration and proliferation, but also a proportion of locally proliferating pericytes becomes the source of cells with a microglial phenotype in the infarct area. Pericytes have recently attracted increasing interest due to their multipotentiality and their emerging role in tissue repair in different organs [11, 23, 40, 43]. Here, we investigate how brain pericytes respond to an ischemic insult. We track the fate of RGS5^+^ pericytes in response to focal ischemic brain injury using a knock-out/knock-in mouse strain where GFP is expressed from the *Rgs5* locus. We confirm previous findings showing that RGS5 is a marker specific for pericytes [8, 10], and thus selectively labels this cell type [39].

In response to ischemia, pericytes are first activated as shown by increased expression of RGS5 and NG2. Both markers are known to be upregulated in activated pericytes and pericytes undergoing angiogenesis [7, 41]. The activation of pericytes is followed by a strong proliferative response demonstrated by BrdU incorporation and by the expression of Ki67. In contrast to our findings, extensive pericyte cell death within 24 h following pericyte constriction in response to stroke has recently been described [28]. It is conceivable that different subtypes of capillary pericytes have divergent fates, and RGS5-expression in pericytes may identify a subpopulation of newborn, proliferating pericytes.

We show that pericytes leave the blood vessels after stroke and this separation from the vascular compartment is associated with a change to an ameboid morphology and migration into the brain parenchyma. Migratory behavior and morphological changes of pericytes have been described following stroke and traumatic brain injury [16, 22, 31] and are associated with disruption of the basement membrane.

Using immunocytochemistry and qPCR, we demonstrate that pericytes acquire a microglial phenotype in response to hypoxic/ischemic conditions, both in vivo and in vitro. GFP^+^ cells that had migrated into the parenchyma acquired an ameboid morphology and expressed several microglia-specific markers, IBA1, CD11b and GAL-3, 1 week after the ischemic insult. Importantly, GFP expression was not detected in the resting microglia in the contralateral *Rgs* ^*gfp/*+^ mouse hemisphere. We did not observe RGS5 upregulation in OGD-stimulated microglial cells derived from *Rgs* ^*gfp/*+^ mice confirming that RGS5 is specifically expressed in pericytes and not upregulated in activated microglia [8, 10]. Furthermore, GFP^+^ pericytes give only rise to a proportion of microglial cells, arguing against an unspecific upregulation of RGS5 in all microglial cells. Similarly, it is extremely unlikely that the reporter becomes ectopically expressed due to deletions in the intronic areas of the gene. Another possibility is that the GFP^+^ cells in fate-mapping studies arise from cell fusion. This is highly unlikely as well as fusion is a rare event [45] and is thus in contrast to the large increase in the number of GFP^+^ cells that we observed.

Consistent with the phenotypic transformation of pericytes in vivo, human brain-derived pericytes exposed to hypoxic conditions in vitro change their morphology, express microglial markers and upregulate mRNA for several microglial genes (*CD11B*, *GAL*-*3*, *IBA1*, *TNFA*, *MHC11*). Taken together, these findings demonstrate the inherent capability of pericytes to acquire a microglial phenotype.

Unlike reported in a recent study [23], RGS5^+^ pericytes do not express markers associated with fibroblasts, such as collagen-IV, fibronectin and fibroblast surface protein and hence do not contribute to scar formation as has been shown for PDGFRβ^+^ pericytes [2, 20, 23]. This suggests that pericytes may either respond differently to different types of injury or in different organs or, more likely, that different subtypes of pericytes have distinctive differentiation capacities.

Interestingly, RGS5^+^ pericyte-derived cells did not express markers of monocytes/macrophages (CD68, CD45). Monocytes and macrophages are bone marrow-derived and infiltrate the mouse brain during ischemia [37]. Even though we observed GFP^+^ pericytes in the bone marrow around the blood vessels, albeit at low frequency, we did not find a significant increase in GFP^+^ cells in response to stroke. We identified a CD11b^+^/CD45^+^ cell population in the bone marrow; however, none of these cells co-expressed GFP. This makes it highly unlikely that RGS5^+^ pericyte-derived microglial cells are bone marrow-derived and have infiltrated the ischemic brain from the blood. This is consistent with findings in *Rgs5* ^*gfp/*+^ bone marrow chimeric mice where no significant contribution of GFP^+^ cells from the bone marrow to the brain was found after stroke [20]. In addition, GFP^+^ cells strongly proliferated in the parenchyma, whereas bone marrow-derived macrophages do not proliferate in the brain [27, 33, 35].

The CD68^+^ cells found in the perivascular space in the brain morphologically resembled pericytes but did not co-label with GFP, indicating that these cells are most likely perivascular macrophages [24, 46]. Indeed, CD68^+^ cells co-expressed CD11b at later stages after the ischemic injury; hence, they are most likely a distinct subpopulation of activated macrophages that is recruited to the injury site [24, 44, 46].

Microglial cells are known to be activated in stroke, adopt an ameboid morphology, have antigen-presenting capacities and the ability to phagocyte cell debris [37]. They are also closely associated with the microvasculature in the brain, indicating a relationship between pericytes and microglia [27]. Interestingly, pericytes have been described as an origin of follicular dendritic cells in lymphoid tissue [34] and phagocyte-like characteristics and CD11b expression were reported in a subpopulation of pericytes, suggesting an immune function for pericytes [5]. It is conceivable that pericytes and microglial cells may share the same ancestor and it has been proposed that they originate from either mesoderm [1, 21, 32, 48] or neuroectoderm [18] during development.

In conclusion, our data support that pericytes are a previously unknown source of microglial cells in brain ischemia. Further studies are warranted to identify the functional role of pericytes in brain ischemia and other pathological conditions.

## References

[1] Alliot F, Godin I, Pessac B (1999). Microglia derive from progenitors, originating from the yolk sac, and which proliferate in the brain. Brain Res Dev Brain Res.

[2] Arimura K, Ago T, Kamouchi M, Nakamura K, Ishitsuka K, Kuroda J, Sugimori H, Ooboshi H, Sasaki T, Kitazono T (2012). PDGF receptor beta signaling in pericytes following ischemic brain injury. Curr Neurovasc Res.

[3] Armulik A, Genove G, Mae M, Nisancioglu MH, Wallgard E, Niaudet C, He L, Norlin J, Lindblom P, Strittmatter K, Johansson BR, Betsholtz C (2010). Pericytes regulate the blood-brain barrier. Nature.

[4] Audoy-Remus J, Richard JF, Soulet D, Zhou H, Kubes P, Vallieres L (2008). Rod-shaped monocytes patrol the brain vasculature and give rise to perivascular macrophages under the influence of proinflammatory cytokines and angiopoietin-2. J Neurosci.

[5] Balabanov R, Washington R, Wagnerova J, Dore-Duffy P (1996). CNS microvascular pericytes express macrophage-like function, cell surface integrin alpha M, and macrophage marker ED-2. Microvasc Res.

[6] Bell RD, Winkler EA, Sagare AP, Singh I, LaRue B, Deane R, Zlokovic BV (2010). Pericytes control key neurovascular functions and neuronal phenotype in the adult brain and during brain aging. Neuron.

[7] Berger M, Bergers G, Arnold B, Hammerling GJ, Ganss R (2005). Regulator of G-protein signaling-5 induction in pericytes coincides with active vessel remodeling during neovascularization. Blood.

[8] Bondjers C, Kalen M, Hellstrom M, Scheidl SJ, Abramsson A, Renner O, Lindahl P, Cho H, Kehrl J, Betsholtz C (2003). Transcription profiling of platelet-derived growth factor-B-deficient mouse embryos identifies RGS5 as a novel marker for pericytes and vascular smooth muscle cells. Am J Pathol.

[9] Chen CW, Montelatici E, Crisan M, Corselli M, Huard J, Lazzari L, Peault B (2009). Perivascular multi-lineage progenitor cells in human organs: regenerative units, cytokine sources or both?. Cytokine Growth Factor Rev.

[10] Cho H, Kozasa T, Bondjers C, Betsholtz C, Kehrl JH (2003). Pericyte-specific expression of Rgs5: implications for PDGF and EDG receptor signaling during vascular maturation. Faseb J.

[11] Crisan M, Yap S, Casteilla L, Chen CW, Corselli M, Park TS, Andriolo G, Sun B, Zheng B, Zhang L, Norotte C, Teng PN, Traas J, Schugar R, Deasy BM, Badylak S, Buhring HJ, Giacobino JP, Lazzari L, Huard J, Peault B (2008). A perivascular origin for mesenchymal stem cells in multiple human organs. Cell Stem Cell.

[12] Deierborg T (2013). Preparation of primary microglia cultures from postnatal mouse and rat brains. Methods Mol Biol.

[13] Dellavalle A, Maroli G, Covarello D, Azzoni E, Innocenzi A, Perani L, Antonini S, Sambasivan R, Brunelli S, Tajbakhsh S, Cossu G (2011). Pericytes resident in postnatal skeletal muscle differentiate into muscle fibres and generate satellite cells. Nat Commun.

[14] Dirnagl U, Becker K, Meisel A (2009). Preconditioning and tolerance against cerebral ischaemia: from experimental strategies to clinical use. Lancet Neurol.

[15] Dore-Duffy P, Cleary K (2011). Morphology and properties of pericytes. Methods Mol Biol.

[16] Dore-Duffy P, Owen C, Balabanov R, Murphy S, Beaumont T, Rafols JA (2000). Pericyte migration from the vascular wall in response to traumatic brain injury. Microvasc Res.

[17] Dulmovits BM, Herman IM (2012). Microvascular remodeling and wound healing: a role for pericytes. Int J Biochem Cell Biol.

[18] Etchevers HC, Vincent C, Le Douarin NM, Couly GF (2001). The cephalic neural crest provides pericytes and smooth muscle cells to all blood vessels of the face and forebrain. Development (Cambridge, England).

[19] Feng J, Mantesso A, De Bari C, Nishiyama A, Sharpe PT (2011). Dual origin of mesenchymal stem cells contributing to organ growth and repair. Proc Natl Acad Sci USA.

[20] Fernandez-Klett F, Potas JR, Hilpert D, Blazej K, Radke J, Huck J, Engel O, Stenzel W, Genove G, Priller J (2013). Early loss of pericytes and perivascular stromal cell-induced scar formation after stroke. J Cereb Blood Flow Metab.

[21] Ginhoux F, Greter M, Leboeuf M, Nandi S, See P, Gokhan S, Mehler MF, Conway SJ, Ng LG, Stanley ER, Samokhvalov IM, Merad M (2010). Fate mapping analysis reveals that adult microglia derive from primitive macrophages. Science (New York, NY).

[22] Gonul E, Duz B, Kahraman S, Kayali H, Kubar A, Timurkaynak E (2002). Early pericyte response to brain hypoxia in cats: an ultrastructural study. Microvasc Res.

[23] Goritz C, Dias DO, Tomilin N, Barbacid M, Shupliakov O, Frisen J (2011). A pericyte origin of spinal cord scar tissue. Science (New York, NY).

[24] Graeber MB, Streit WJ, Kreutzberg GW (1989). Identity of ED2-positive perivascular cells in rat brain. J Neurosci Res.

[25] Gregersen R, Christensen T, Lehrmann E, Diemer NH, Finsen B (2001). Focal cerebral ischemia induces increased myelin basic protein and growth-associated protein-43 gene transcription in peri-infarct areas in the rat brain. Exp Brain Res Experimentelle Hirnforschung.

[26] Gregersen R, Lambertsen K, Finsen B (2000). Microglia and macrophages are the major source of tumor necrosis factor in permanent middle cerebral artery occlusion in mice. J Cereb Blood Flow Metab.

[27] Guillemin GJ, Brew BJ (2004). Microglia, macrophages, perivascular macrophages, and pericytes: a review of function and identification. J Leukoc Biol.

[28] Hall CN, Reynell C, Gesslein B, Hamilton NB, Mishra A, Sutherland BA, O’Farrell FM, Buchan AM, Lauritzen M, Attwell D (2014). Capillary pericytes regulate cerebral blood flow in health and disease. Nature.

[29] Humphreys BD, Lin SL, Kobayashi A, Hudson TE, Nowlin BT, Bonventre JV, Valerius MT, McMahon AP, Duffield JS (2010). Fate tracing reveals the pericyte and not epithelial origin of myofibroblasts in kidney fibrosis. Am J Pathol.

[30] Inacio AR, Ruscher K, Leng L, Bucala R, Deierborg T (2011). Macrophage migration inhibitory factor promotes cell death and aggravates neurologic deficits after experimental stroke. J Cereb Blood Flow Metab.

[31] Jeynes B (1985). Reactions of granular pericytes in a rabbit cerebrovascular ischemia model. Stroke: J Cereb Circ.

[32] Kierdorf K, Erny D, Goldmann T, Sander V, Schulz C, Perdiguero EG, Wieghofer P, Heinrich A, Riemke P, Holscher C, Muller DN, Luckow B, Brocker T, Debowski K, Fritz G, Opdenakker G, Diefenbach A, Biber K, Heikenwalder M, Geissmann F, Rosenbauer F, Prinz M (2013). Microglia emerge from erythromyeloid precursors via Pu.1- and Irf8-dependent pathways. Nat Neurosci.

[33] Kokovay E, Li L, Cunningham LA (2006). Angiogenic recruitment of pericytes from bone marrow after stroke. J Cereb Blood Flow Metab.

[34] Krautler NJ, Kana V, Kranich J, Tian Y, Perera D, Lemm D, Schwarz P, Armulik A, Browning JL, Tallquist M, Buch T, Oliveira-Martins JB, Zhu C, Hermann M, Wagner U, Brink R, Heikenwalder M, Aguzzi A (2012). Follicular dendritic cells emerge from ubiquitous perivascular precursors. Cell.

[35] Krueger M, Bechmann I (2010). CNS pericytes: concepts, misconceptions, and a way out. Glia.

[36] Lalancette-Hebert M, Swarup V, Beaulieu JM, Bohacek I, Abdelhamid E, Weng YC, Sato S, Kriz J (2012). Galectin-3 is required for resident microglia activation and proliferation in response to ischemic injury. J Neurosci.

[37] Lambertsen KL, Deierborg T, Gregersen R, Clausen BH, Wirenfeldt M, Nielsen HH, Dalmau I, Diemer NH, Dagnaes-Hansen F, Johansen FF, Keating A, Finsen B (2011). Differences in origin of reactive microglia in bone marrow chimeric mouse and rat after transient global ischemia. J Neuropathol Exp Neurol.

[38] Mendez-Ferrer S, Lucas D, Battista M, Frenette PS (2008). Haematopoietic stem cell release is regulated by circadian oscillations. Nature.

[39] Nisancioglu MH, Mahoney WM, Kimmel DD, Schwartz SM, Betsholtz C, Genove G (2008). Generation and characterization of rgs5 mutant mice. Mol Cell Biol.

[40] Özen I, Boix J, Paul G (2012) Perivascular mesenchymal stem cells in the adult human brain: a future target for neuroregeneration? Clin Transl Med 1(1:30). doi:10.1186/2001-1326-1-30 10.1186/2001-1326-1-30 PMC3561038 23369339

[41] Ozerdem U, Grako KA, Dahlin-Huppe K, Monosov E, Stallcup WB (2001). NG2 proteoglycan is expressed exclusively by mural cells during vascular morphogenesis. Dev Dyn.

[42] Ozerdem U, Monosov E, Stallcup WB (2002). NG2 proteoglycan expression by pericytes in pathological microvasculature. Microvasc Res.

[43] Paul G, Ozen I, Christophersen NS, Reinbothe T, Bengzon J, Visse E, Jansson K, Dannaeus K, Henriques-Oliveira C, Roybon L, Anisimov SV, Renstrom E, Svensson M, Haegerstrand A, Brundin P (2012). The adult human brain harbors multipotent perivascular mesenchymal stem cells. PLoS One.

[44] Perego C, Fumagalli S, De Simoni MG (2011). Temporal pattern of expression and colocalization of microglia/macrophage phenotype markers following brain ischemic injury in mice. J Neuroinflam.

[45] Piquer-Gil M, Garcia-Verdugo JM, Zipancic I, Sanchez MJ, Alvarez-Dolado M (2009). Cell fusion contributes to pericyte formation after stroke. J Cereb Blood Flow Metab.

[46] Rajantie I, Ilmonen M, Alminaite A, Ozerdem U, Alitalo K, Salven P (2004). Adult bone marrow-derived cells recruited during angiogenesis comprise precursors for periendothelial vascular mural cells. Blood.

[47] Schlingemann RO, Rietveld FJ, de Waal RM, Ferrone S, Ruiter DJ (1990). Expression of the high molecular weight melanoma-associated antigen by pericytes during angiogenesis in tumors and in healing wounds. Am J Pathol.

[48] Schulz C, Gomez Perdiguero E, Chorro L, Szabo-Rogers H, Cagnard N, Kierdorf K, Prinz M, Wu B, Jacobsen SE, Pollard JW, Frampton J, Liu KJ, Geissmann F (2012). A lineage of myeloid cells independent of Myb and hematopoietic stem cells. Science (New York, NY).

[49] Sims DE (1991). Recent advances in pericyte biology—implications for health and disease. Can J Cardiol.

[50] Tang W, Zeve D, Suh JM, Bosnakovski D, Kyba M, Hammer RE, Tallquist MD, Graff JM (2008). White fat progenitor cells reside in the adipose vasculature. Science (New York, NY).

